# Biochemical Studies in Perfundates and Homogenates of Isolated Porcine Kidneys after Flushing with Zinc or Zinc–Prolactin Modified Preservation Solution Using a Static Cold Storage Technique

**DOI:** 10.3390/molecules26113465

**Published:** 2021-06-07

**Authors:** Aneta Ostróżka-Cieślik, Barbara Dolińska, Florian Ryszka

**Affiliations:** 1 Department of Pharmaceutical Technology, Faculty of Pharmaceutical Sciences in Sosnowiec, Medical University of Silesia, Kasztanowa 3, 41-200 Sosnowiec, Poland; bdolinska@sum.edu.pl; 2 “Biochefa” Pharmaceutical Research and Production Plant, Kasztanowa 3, 41-200 Sosnowiec, Poland; f.ryszka@biochefa.pl

**Keywords:** zinc, prolactin, Biolasol, perfusion, preservation, renal

## Abstract

Zinc is an effective anti-inflammatory and antioxidant trace element. The aim of this study was to analyse the protective effect of zinc and zinc–prolactin systems as additives of preservation solutions in the prevention of nephron damage caused during ischemia. The study used a model for storing isolated porcine kidneys in Biolasol^®^. The solution was modified with the addition of Zn at a dose of 1 µg/L and Zn: 1 µg/L with prolactin (PRL): 0.1 µg/L. After 2 h and 48 h of storage, the levels of alanine aminotransferase, aspartate aminotransferase, lactate dehydrogenase, sodium, potassium, creatinine and total protein were determined. Zinc added to the Biolasol^®^ composition at a dose of 1 µg/L showed minor effectiveness in the protection of nephrons. In turn, Zn^2+^ added to Biolasol + PRL (PRL: 0.1 µg/L) acted as a prolactin inhibitor. We do not recommend the addition of Zn(II) (1 µg/L) and Zn(II) (1 µg/L) + PRL (0.1 µg/L) to the Biolasol solution.

## 1. Introduction

Zinc is a trace element that regulates many biochemical processes in the human body. It affects proper development, participates in the synthesis of hormones (testosterone, insulin), is a cofactor of active thymulin (ZnFTS, zinc-facteur-timique-serique) released by thymic cells, and participates in the stabilization of cell membranes. It regulates the functioning of the immune and nervous systems. In humans, 10% of proteins have zinc-binding sites. In turn, 85–90% of Zn^2+^ in the body is closely related to proteins, including metalloenzymes and metalloproteins. It is a component of enzymes, including DNA and RNA polymerases. It is a part of superoxide dismutase, carbonic anhydrase, lactate dehydrogenase, malate dehydrogenase and glutamate dehydrogenase. As a component of superoxide dismutase, it reduces the production of reactive oxygen species and protects against oxidative damage. In turn, being part of carbonic anhydrase, it affects the acid–base balance, regulating it in the lungs and renal tubules [1,2,3,4,5]. Two mechanisms of zinc antioxidant activity have been confirmed, i.e., protection of sulfhydryl groups of proteins and inhibition of factors that intensify the production of oxygen free radicals. Zinc is antagonistic to the transition metals copper and iron, which exhibit redox activity. It induces the activity of metallothioneins (MTs, low molecular weight metal-binding proteins) in the organs that remove oxidants [2,6]. Its normal concentration in the blood serum ranges from 8.4 to 22.9 µmol/L; 50% of zinc is deposited in the cell cytoplasm, 30–40% in the nucleus, and 10% in the cell membrane [7]. Zinc participates in the regulation of intra- and extracellular pathways involved in cell proliferation, differentiation, development, apoptosis, and transformation [8].

The aim of this research was to analyse the protective effect of zinc as a component of Biolasol in the prevention of nephron damage caused during ischemia. The solution was modified with the addition of Zn at a dose of 1 µg/L and Zn: 1 µg/L with prolactin (PRL): 0.1 µg/L. These doses were chosen based on earlier studies and our pilot studies. The introduction of PRL to the composition of the Biolasol solution was dictated by our previous research experience. We confirmed that prolactin reduced markers that indicate loss of quality of isolated porcine kidneys during perfusion, storage and reperfusion. This hormone can enhance the antioxidant effect of zinc [9,10]. The components of the preservation solutions can have cumulative effects and show a synergistic effect. Moreover, zinc plays an important role in the structural integrity of PRL [11]. The study used a model of storing isolated porcine kidneys in the Biolasol/modified Biolasol solution for static cold storage, which is the standard method of organ preservation. Biolasol includes components supporting the maintenance of structural and functional graft integrity [12,13].

## 2. Results

**Figure 1:**
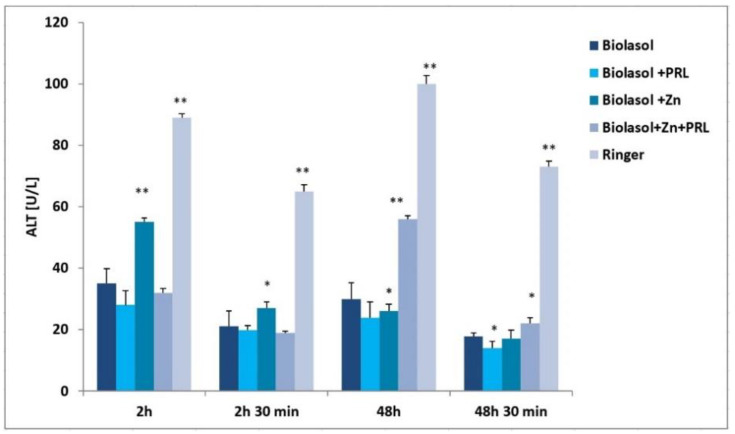
ALT activity in the collected perfusates and reperfusates in model of storing isolated porcine kidneys. The values are expressed as mean ± SEM. Data were analysed by one-way ANOVA and Bonferroni post hoc tests; n = 10; * *p* < 0.05; ** *p* < 0.01 compared to the control group (Biolasol).

**Figure 2:**
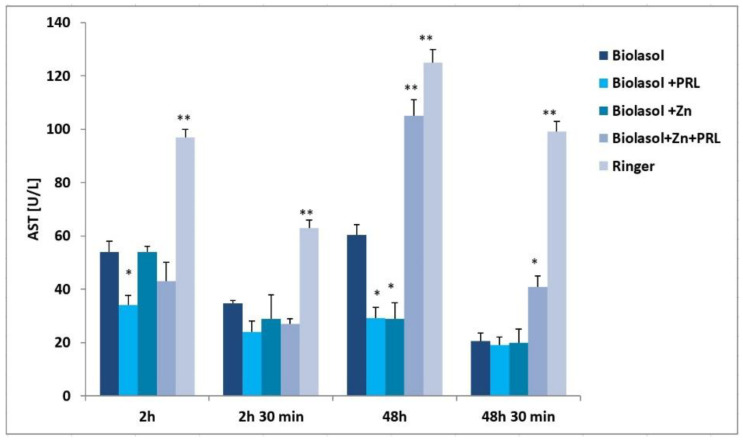
AST activity in the collected perfusates and reperfusates in model of storing isolated porcine kidneys. The values are expressed as mean ± SEM. Data were analysed by one-way ANOVA and Bonferroni post hoc tests; n = 10; * *p* < 0.05; ** *p* < 0.01 compared to the control group (Biolasol).

**Figure 3:**
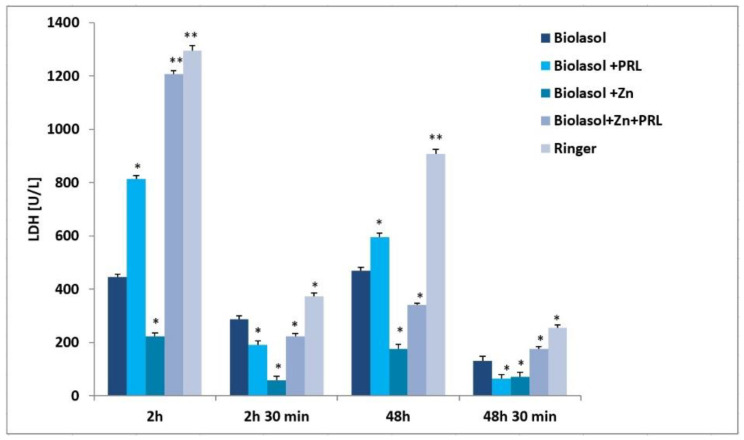
LDH activity in the collected perfusates and reperfusates in model of storing isolated porcine kidneys. The values are expressed as mean ± SEM. Data were analysed by one-way ANOVA and Bonferroni post hoc tests; n = 10; * *p* < 0.05; ** *p* < 0.01 compared to the control group (Biolasol).

Nephrocyte cytolysis was determined by measuring the activity of aspartate aminotransferase (AST), alanine aminotransferase (ALT) and lactate dehydrogenase (LDH) (Figure 1, Figure 2 and Figure 3). Ischemia-reperfusion injury occurred in all the study groups. After 2 h of kidney preservation, high levels of markers were determined, which indicates disorders in the proper functioning of cell membranes. After 48 h of storage (vs. 2 h), a decrease in ALT activity was observed. The greatest decrease in this parameter (53%) was found in the Biolasol + Zn group. ALT activity in the Biolasol + PRL (48 h) group was 21% less than in Biolasol (48 h) (*p* < 0.05). On the other hand, the activity of ALT in the group Biolasol + PRL + Zn (48 h) was lower by 13% compared to Biolasol (48 h) (*p* = ns). After 48 h, the AST activity decreased by 52% in the Biolasol + PRL vs. Biolasol group and by 51% in the Biolasol + Zn vs. Biolasol group (*p* < 0.05). LDH activity decreased by 62% in the Biolasol + Zn group (vs. Biolasol) (*p* < 0.05).

A significant decrease in the activity of indicator enzymes in the individual groups was found after 48 h 30 min of reperfusion, which suggests regression of damage and a protective effect of the analysed solutions on the kidneys. After this time, the lowest marker activity values were found in the Biolasol + PRL group (ALT: 14 ± 1 U/L; AST: 19 ± 2 U/L; LDH: 65 ± 11 (*p* < 0.05), and the highest in the Biolasol + Zn + PRL group (ALT: 22 ± 2 U/L; AST: 41 ± 4 U/L, LDH: 175 ± 10 (*p* < 0.05).

**Figure 4:**
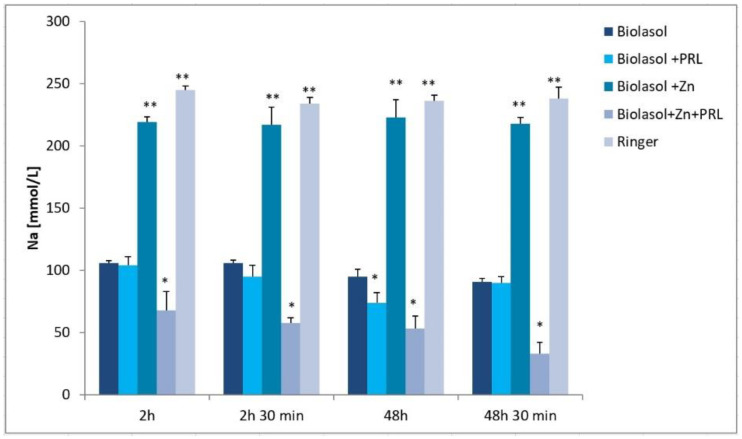
Sodium concentrations in the collected perfusates and reperfusates in model of storing isolated porcine kidneys. The values are expressed as mean ± SEM. Data were analysed by one-way ANOVA and Bonferroni post hoc tests; n = 10; * *p* < 0.05; ** *p* < 0.01 compared to the control group (Biolasol).

**Figure 5:**
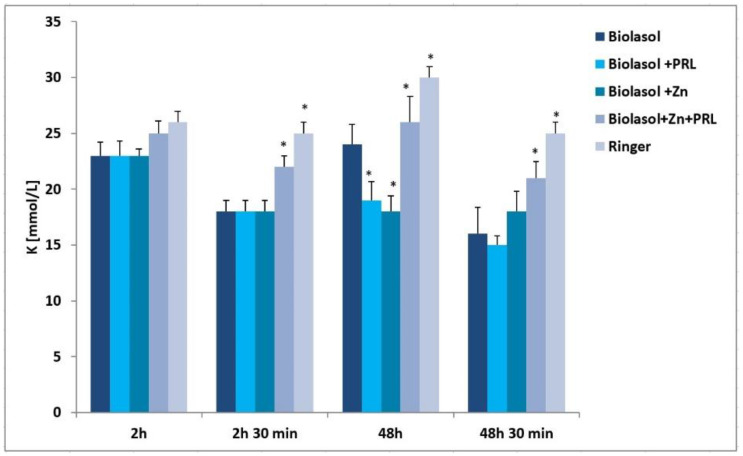
Potassium concentrations in the collected perfusates and reperfusates in model of storing isolated porcine kidneys. The values are expressed as mean ± SEM. Data were analysed by one-way ANOVA and Bonferroni post hoc tests; n = 10; * *p* < 0.05 compared to the control group (Biolasol).

Oxygen deficit inhibits the Na^+^/K^+^-ATPase ion pump in the cell membrane and increases intracellular metabolic acidosis. Disruption of the transmembrane ion gradient results in abnormal graft function. High concentrations of sodium ions were observed in the perfusates/reperfusates of the Biolasol + Zn group (2 h/219 ± 6 mmol/L, 2 h 30 min/217 ± 8 mmol/L, 48 h/223 ± 14 mmol/L, 48 h 30 min/218 ± 10 mmol/L), which were significantly higher compared to the Biolasol, Biolasol + PRL and Biolasol + Zn + PRL groups (*p* < 0.01) (Figure 4). In turn, the lowest concentration of sodium ions was determined in the samples collected in the Biolasol + Zn + PRL group (2 h/68 ± 3 mmol/L, 2 h 30 min/58 ± 5 mmol/L, 48 h/53 ± 5 mmol/L, 48 h 30 min/33 ± 9 mmol/L) (*p* < 0.05). In the same group (Biolasol + Zn + PRL), the concentration of K^+^ ions was significantly higher (2 h/25 ± 2 mmol/L, 2 h 30 min/22 ± 1 mmol/L, 48 h/26 ± 2 mmol/L, 48 h 30 min/21 ± 1 mmol/L) (*p* < 0.05). The lowest K^+^ concentration was found in the Biolasol + PRL group after 48 h 30 min (15 ± 1 mmol/L). The lowest concentration of K^+^ after 48-h graft storage was found in the Biolasol + Zn group (decrease by 25% vs. Biolasol/48 h) (*p* < 0.05) (Figure 5).

**Figure 6:**
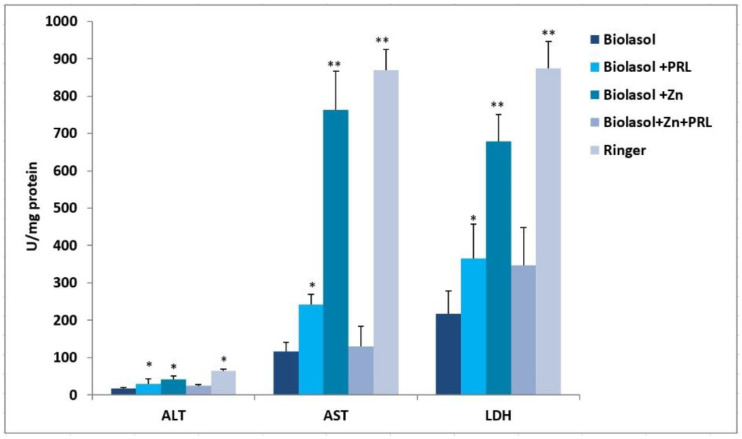
Activity of biochemical markers in the kidney homogenates in model of storing isolated porcine kidneys. The values are expressed as mean ± SEM. Data were analysed by one-way ANOVA and Bonferroni post hoc tests; n = 10; * *p* < 0.05; ** *p* < 0.01 compared to the control group (Biolasol).

**Figure 7:**
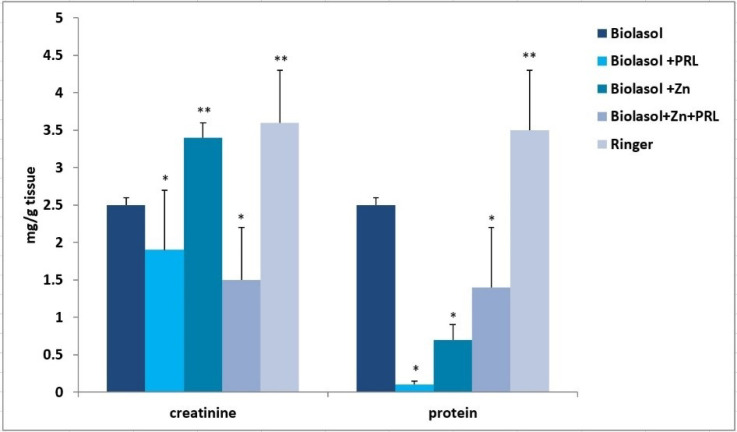
Creatinine and protein concentrations in the kidney homogenates in model of storing isolated porcine kidneys. The values are expressed as mean ± SEM. Data were analysed by one-way ANOVA and Bonferroni post hoc tests; n = 10; * *p* < 0.05; ** *p* < 0.01 compared to the control group (Biolasol).

Figure 6 shows the effect of Biolasol/modified Biolasol on the biochemical parameters (ALT, AST, LDH) in a renal tissue homogenate. Significant increases in ALT, AST and LDH activity were found in the Biolasol + Zn group compared to the Biolasol, Biolasol + PRL and Biolasol + Zn + PRL groups (*p* < 0.01). Rinsing kidneys with Biolasol + Zn increased the creatinine concentrations in the renal tissue homogenates (3.4 ± 0.2 mg/g tissue, *p* < 0.01) (Figure 7). The protein concentration was 0.7 ± 0.2 mg/g tissue, *p* < 0.05). Figure 1, Figure 2 and Figure 3 show the activity of ALT, AST and LDH in the collected perfusates. Figure 6 shows the activities of the above enzymes, which were determined in the supernatants of homogenates (cytoplasmic fraction). A clear increase in the activities of ALT, AST, LDH in the Biolasol + Zn group (Figure 6) indicates the activation of cell disruption processes after 48 h 30 min.

## 3. Discussion

Biolasol is a solution developed in Poland for ex vivo perfusion and the preservation of kidney, liver, pancreas and heart. The osmotic pressure of the fluid is 330 mOsm/L, pH = 7.4. The total concentration of Na^+^ (105 mmol/L) and K^+^ (10 mmol/L) points to an extracellular solution. Biolasol supports the structural and functional integrity of grafts and minimizes ischemia–reperfusion injury. The solution contains electrolytes, osmotically and oncotically active substances, buffering systems, substances preventing cellular acidosis, which are a source of energy, and antioxidants. Dextran 70 kDa allows the fluid to be moved from the interstitial space to the intravascular space, which minimizes cell swelling. In addition, it improves the capillary circulation in the graft, preventing the aggregation of blood cells. Glucose is a substrate for ATP resynthesis. Trisodium citrate binds calcium ions that are involved in the blood coagulation process. In addition, it maintains the acid–base balance of the intracellular environment. EDTA complexes cations of multivalent metals (including iron ions) minimizing free radical damage. Magnesium fumarate as a source of Mg^2+^ ions affects the maintenance of the structural and functional integrity of the lipid bilayer. Sodium bicarbonate acts as a buffer that maintains the acid–base balance of the transplanted organ [14,15]. Budziński et al. [16] analysed the effectiveness of Biolasol in an animal model of transgenic pigs with the transferred human gene: α1,2-fucosyltransferase (group I and II), α-galactosidase (III), combined α1,2-fucosyltransferase/α-galactosidase transgene (IV), and livers without modification (V). The isolated livers were perfused and stored in Biolasol for 24 h. In the collected perfundates and homogenates, they analysed IL-6 (interleukin-6) concentration. Based on the obtained results, they revealed hepatoprotective effects of Biolasol. Cierpka’s team [11] compared the effectiveness of Biolasol and UW in the animal model of perfusion and preservation of the isolated kidney of the Polish Large White pig. They analysed changes in the concentration of enzyme markers (ALT, AST, LDH) in the collected perfundates, specimens collected for histopathological examinations and mean survival time of the recipient after autotransplantation. They found non-inferiority of the effectiveness of Biolasol in relation to the reference solution, i.e., UW. Dolińska et al. [13] compared the effectiveness of Biolasol and HTK in maintaining normal homeostasis of stored porcine kidneys. The activity of released indicator enzymes (ALT, AST, LDH) and concentrations of sodium, potassium and magnesium ions were subjected to analysis. Biolasol and HTK protect kidneys against ischemic damage effectively. However, Biolasol provided more optimal homeostasis, which may suggest its better nephroprotective properties. Jóźwik et al. [17] transplanted 42 human kidneys, which were previously stored by simple hypothermia in Biolasol and the reference solution—UW. They found that delayed graft function occurred in both groups of patients (38% of cases in the Biolasol group vs. 33% in the UW group, *p* = ns). Therefore, an average of 2.25 patients whose kidneys were rinsed with Biolasol and 1.86 patients whose grafts were rinsed with UW were subjected to hemodialysis. Creatinine concentration was determined in patients after the transplantation, and it was found that after 7, 30 and 60 days, the value was 4.64 mg/dL, 1.75 mg/dL, 1.7 mg/dL (Biolasol group) and 3.2 mg/dL, 1.53 mg/dL, 1.62 mg/dL (UW group), respectively. The effectiveness of the used solutions was comparable.

Zinc is a stable divalent cation that does not directly undergo redox reactions. The flow of zinc ions into and out of the cell is possible owing to the presence of its importers, exporters and metallothioneins. The most important zinc transporters include proteins from the ZnT (SLC30, Soluble Carrier 30) and Zip (SLC39, Soluble Carrier 39) families, which show the opposite effect [18]. The kidneys are the organs most involved in maintaining zinc homeostasis. Zn^2+^ balance is achieved by renal reabsorption. The filtered zinc ions are reabsorbed along the nephron, near the proximal renal tubules [19]. ZnT1 facilitates zinc reabsorption in the renal epithelial cells. ZnT2 and ZnT4 transport the excess of cytosolic zinc to the secretory vesicles [7,19].

The conducted research suggests that zinc influences the ischemia–reperfusion injury (IRI) of kidneys in an animal model. Moslemi et al. [20] investigated the effect of this trace element on IRI-induced renal failure in rats. The rodents were administered zinc sulphate at a dose of 10 mg/kg/day for 5 days and it was found that Zn^2+^ ions improved renal function. Abdallah et al. [21] observed the effect of Zn^2+^ supplementation (dose of 50 mg/kg; 24 h and 30 min before IRI) on modulation of endoplasmic reticulum (ER) stress, reduced inflammation, and low expression of the autophagy-related proteins Beclin 1 and LAMP-2 (lysosome-associated membrane protein 2) after IRI. The research was carried out in a model of bilateral renal ischemia in rats with subsequent reperfusion. Zinc supplementation (10 mg/kg/day; 3 weeks) also improved renal function in the ischemia/reperfusion injury model in ovariectomized rats. An improvement in serum blood urea nitrogen and creatinine concentrations was found [22]. O’Kane et al. [23] investigated the renoprotective effect of Zn^2+^ (doses of 0.1, 0.5, 1.0, 2.5, 10.0 mg/kg) preconditioning in a clinically relevant sheep model of IRI. Zinc upregulates hypoxia-inducible factor proteins. Mazaheri et al. [24] found that zinc (30 mg/kg) reduced creatinine concentrations and minimized damage to kidney tissue in IRI-induced rats. Hegenauer et al. [25] also showed that intravenous administration of Zn^2+^ (5 mg ZnCl_2_/kg) 30 min before the period of warm ischemia significantly improved renal function in a rabbit. In turn, administration of ZnCl_2_ at a dose of 10 mg/kg 60 min before IRI in a rat model decreased creatinine and urea concentrations and the activity of histological markers [26].

The activation of anaerobic cell metabolism and ATP hydrolysis under hypoxic conditions causes a significant decrease in the cytoplasmic pH, activation of the Na^+^/H^+^ antiporter and an increase in the Na^+^ concentration. Mechanisms counteracting the excessive accumulation of hydrogen ions in the cytoplasm are activated. This is mainly done via the Na^+^/H^+^ pump. Na^+^/H^+^ antiporters remove hydrogen ions from inside the cell, while taking sodium ions [27]. Rinsing the isolated porcine kidneys with Biolasol + Zn + PRL had the greatest impact on the development of hyponatremia (Figure 4). There was probably the influx of Na^+^ ions and the Zn^2+^ complex with prolactin inside the cell, according to the sodium/amino acid cotransport mechanism [28]. Coupling to amino acid uptake is an important mechanism for Na^+^ entry into cells [29]. Zn^2+^ ions form strong bonds with sulphur ligands contained in the amino acid protein sequence, including prolactin [30]. The ability of histidine, contained in the PRL structure, to bind Zn^2+^ ions (potential binding sites: H27A, H30A and H180A) was confirmed [12,31]. The association constant of Zn^2+^ ion binding to bovine PRL is 2 × 10^5^ M^−1^ at pH 6.5 [12]. In turn, rinsing the kidneys with Biolasol + Zn resulted in the occurrence of hypernatremia, which suggests that Zn^2+^ can influence the activity of ion channels in intracellular acidosis. Activation of the channels increased the Na^+^ concentration outside the cell as a result of cell depolarization with simultaneous Zn^2+^ influx into the cell [32]. Zinc acts contrary to sodium [33]. Is suggested that a sodium-dependent mechanism may control renal tubular reabsorption of zinc [34]. Dünkelberg et al. [33] found that sodium may influence the zinc concentration by upregulation of ZIP10 expression.

The optimal electrolyte balance was achieved by rinsing the kidneys with Biolasol + PRL. This confirms the thesis of Ibarra et al. [35] that prolactin is a natriuretic hormone that interacts with the renal dopamine system. PRL inhibits the activity of Na^+^/K^+^-ATPase of proximal tubules. The high structural stability of PRL was confirmed at pH of approximately 6.5 [12].

ALT is present in the cytosolic fraction and its high activity in perfusates/reperfusates indicates damage to the plasma membranes. AST is found in the cytosol and in the mitochondrial fraction. Its high activity indicates disruption of the internal cell structures. LDH, in turn, is a marker of the threshold of anaerobic changes, especially anaerobic glycolysis. Their release into the extracellular space indicates a violation of the structural integrity of cell membranes. We compared the activities of ALT, AST and LDH in the perfundates and homogenates of the analysed groups. Biolasol reduced kidney injury more effectively on its own without additives. Biolasol significantly inhibits the cytolysis of kidney cells. Biolasol solution modified with Zn^2+^ reduced kidney injury concerning AST, ALT and LDH release after 48 h cold ischemic. The use of Biolasol + Zn did not inhibit the activity of indicator enzymes in the renal tissue homogenates. It also increased the creatinine content in cells compared to the Biolasol, Biolasol + PRL, Biolasol + Zn + PRL groups. The lowest protein content in cells was observed in the kidney homogenates washed with Biolasol + PRL. It can be assumed that 0.1 µg/L PRL improves the behaviour of metabolic pathways and reduces the cell/tissue demand for energy [10,36]. Other studies also confirm the protective effect of sex hormones on nephrons in the period of ischemia [37,38,39,40]. The addition of 1 µg/L Zn^2+^ to the modified Biolasol + PRL solution (group B3) caused a significant increase in the activity of enzymes in the kidney homogenates. We suggest, like Brandão et al. [41], that zinc is an inhibitor of prolactin, reduces its activity and removes its protective effect. Zinc plays an important role in the in vivo regulation of prolactin release. Treatment with zinc (50 mg/day) lowers serum prolactin concentration in hemodialysis uremic patients [42].

We found a minor protective effect of zinc added to Biolasol at a dose of 1 µg/L on nephrons. This is probably because the high intracellular Zn^2+^ concentration induces its pro-oxidative properties and influences the conditions of oxidative stress [43]. Free intracellular zinc may cause changes in the permeability of the mitochondrial membrane and the release of cytochrome c [5]. It was concluded that zinc is a potent inducer of heat shock protein (HSP70) activator, during liver cold preservation in rats [44] and may induce cell apoptosis [45]. Oxidative stress and HSP70 can promote the activate of MMPs/zinc-containing enzymes, which are part of the mechanism of injury of preserved organs. MMPs do play a role in damage mitochondrial DNA and increasing mitochondrial membrane permeability, leading in turn to mitochondrial dysfunction and ROS generation [46]. The low effectiveness of Zn^2+^ was confirmed by Ogawa and Mimura’s research [47]. Rats were administered the trace element by intraperitoneal injection (dose of 20 mg/kg) 24 h before the ischemia-reperfusion procedure. Zinc showed antioxidant activity due to metallothionein induction, which had little effect on nephron protection.

So far, only one study has been carried out on the effectiveness of zinc as a component of a renal perfusion and preservation solution. Singh et al. [48] examined the protective effect of the University of Wisconsin (UW) solution modified with zinc-N-acetylcysteine chelate (0.3 mM/19.6 mg–30 mM/1.96 g Zn) on kidneys in NRK-52E cells. Zinc has been delivered intracellularly using NAC as a chelator. They found that the ZnNAC system (max. effect 1 mM/65.4 mg −10 mM/0.654 g Zn) was a strong antioxidant and DNase I endonuclease inhibitor. Its presence in the UW solution composition decreased the activity of caspase-3 and the expression of EndoG (endonuclease G). Authors suggest that ZnNAC may act by inhibiting ROS, at the caspases, by acting on endonucleases and mechanisms of endonuclease interactions. The results of these studies indicate that caspase inhibitors provide partial protection from ischemic injury during kidney preservation. When analysing our results and those obtained by other authors, it can be assumed that the mechanism of zinc action in kidneys is more complex and dose-dependent [19,49,50]. It has also been suggested that different zinc effectiveness may be due to its use in various animal species. According to the international guidelines, the cytoprotective efficacy of drugs found in small animals (mice, rats, rabbits) should be confirmed in large animals [51]. We based our research on the therapeutic efficacy of zinc as a component of the preservation solution intended for the abdominal parenchymal organs in an isolated porcine kidney model, which had not been tested before.

In summary: zinc added to the Biolasol composition at a dose of 1 µg/L showed minor effectiveness in the protection of nephrons. In turn, Zn^2+^ added to Biolasol + PRL (PRL: 0.1 µg/L) acted as a prolactin inhibitor. The research on modifying the Biolasol solution should be continued. More research is essential to elucidating the role relating to zinc (his dose) in the efficiency of preservative solution.

## 4. Materials and Methods

### 4.1. Preservation Solution

We used Biolasol solution (“FZNP” Biochefa, Sosnowiec, Poland). The composition and functions of the individual components are shown in Table 1. Ringer solution was from Baxter Sp. z o.o, Poland (sodium chloride 8.6 g/L, potassium chloride 0.3 g/L, calcium chloride 0.33 g/L). Zinc acetate [Zn(CH_3_COO)_2_] was from POCh S.A Gliwice, Poland. Pig prolactin was from “FZNP” Biochefa, Sosnowiec, Poland. All substances used in the study were of analytical grade.

### 4.2. Animals

Forty kidneys from 20 Polish Large White pigs, weighing 90–110 kg, aged 175–180 days, were included in the study. The animals were slaughtered at the Meat Plant H.A.M in Radzionków/Poland in a special room using 220 V electricity.

### 4.3. Ethical Issues

The research was conducted with the consent of the II Local Ethics Commission for Animal Experiments in Cracow (No. 1046/2013) and in accordance with the European Union regulations (Directive 86/609 CEE) on the protection of animals during slaughter or killing.

### 4.4. Kidney Procurement and Experimental Groups

The kidneys were collected according to the previously described procedure [52]. The grafts were randomly assigned to 4 groups and preserved by static cold storage.

#### 4.4.1. Group A, Control

Perfusion, preservation and * reperfusion of kidneys in the Biolasol solution/control; n = 10 kidneys;

#### 4.4.2. Group B1

Perfusion, preservation and * reperfusion of kidneys in the Biolasol solution modified with PRL (0.1 µg/L); n = 10 kidneys;

#### 4.4.3. Group B2

Perfusion, preservation and * reperfusion of kidneys in the Biolasol solution modified with Zn^2+^ (1 µg/L/elemental zinc); n = 10 kidneys;

#### 4.4.4. Group B3

Perfusion, preservation and * reperfusion of kidneys in the Biolasol solution modified with Zn^2+^ (1 µg/L/elemental zinc) and PRL (0.1 µg/L); n = 10 kidneys.

#### 4.4.5. Group C

Perfusion, preservation and * reperfusion of kidneys in the Ringer’s solution/sham; n = 10 kidneys.

* reperfusion—flushing the graft after 48 h of preservation with the same solution that was used for previous perfusion and preservation.

### 4.5. Experimental Protocol

The isolated porcine kidneys were placed in 500 mL of Biolasol (Group A), Ringer (Group C) or modified Biolasol (Groups B1, B2, B3) chilled to 4 °C and transported in thermostable containers (4–6 °C) to the Biochefa FZNP laboratory. The grafts were stored under simple hypothermia for 2 h. The kidneys were then cannulated (catheter Nelaton CH08, ConvaTec, Deeside, United Kingdom) and perfused (pressure of 73.5 mmHg H_2_O) ensuring a continuous flow of solution stream. Perfusate samples were collected into Eppendorf tubes from the renal vein at two time points: 0 and 30 min of perfusion. After 30 min, the kidneys were stored under simple hypothermia for 48 h (acceptable cold ischemic time for the kidney). After this time, renal reperfusion was performed. The test samples were taken at 0 and 30 min of reperfusion. The collected samples (after perfusion and reperfusion) were centrifuged at 3000 rpm for 15 min at T = 4 °C and stored at T = −20 °C until the tests were completed. Then, the biochemical markers of renal function were determined. Diagnostic tests were applied, allowing for indirect assessment of renal function [10,52,53,54]. The graft samples for biochemical analysis in kidney homogenates were collected after 48 h 30 min.

### 4.6. Determination of Alanine Aminotransferase

The activity of alanine aminotransferase (ALT) was determined using reagent kits (bioMérieux, Lyon, France) at 340 nm (linearity: 0–500 U/L) and expressed as U/L. The analyses were performed in accordance with the manufacturer’s instructions.

### 4.7. Determination of Aspartate Aminotransferase

The activity of aspartate aminotransferase (AST) was determined using reagent kits (bioMérieux, Lyon, France) at 340 nm (linearity: 0–500 U/L) and expressed as U/L. The analyses were performed in accordance with the manufacturer’s instructions.

### 4.8. Determination of Lactate Dehydrogenase Activity

The activity of lactate dehydrogenase (LDH) was determined using reagent kits (bioMérieux, Lyon, France) at 340 nm (linearity: 1000 U/L) and expressed as U/L. The analyses were performed in accordance with the manufacturer’s instructions.

### 4.9. Determination of Sodium Concentration

Sodium concentration was determined using reagent kits (Pointe Scientific INC, Marseille, France) at 405 nm (linearity: 0–300 mmol/L) and expressed as mmol/L. The analyses were performed in accordance with the manufacturer’s instructions.

### 4.10. Determination of Potassium Concentration

Potassium concentration was determined using reagent kits (Pointe Scientific INC, Marseille, France) at 600 nm (linearity: 0–50 mmol/L) and expressed as mmol/L. The analyses were performed in accordance with the manufacturer’s instructions.

### 4.11. Determination of Creatinine Concentration

Creatinine concentration was determined using reagent kits (Pointe Scientific INC, Marseille, France) at 490 nm (linearity: 0–25 mg/dL) and expressed as mg/dL. The analyses were performed in accordance with the manufacturer’s instructions.

### 4.12. Determination of Total Protein Concentration

Total protein concentration was determined using reagent kits (Pointe Scientific INC, Marseille, France) at 540 nm (linearity: 1 to 15 mg/dL) and expressed as mg/dL. The analyses were performed in accordance with the manufacturer’s instructions.

### 4.13. Apparatus

A Marcel S330 spectrophotometer (Marcel, Poland) was used for biochemical tests. The photometric accuracy of the spectrophotometer was ±0.005 Abs.

### 4.14. Biochemical Analysis in Kidney Homogenates

Kidney samples were collected after completion of reperfusion (48 h 30 min). The samples were homogenized in chilled 0.1 M phosphate buffer (pH = 7). Biochemical determinations were performed in supernatants obtained by centrifuging the homogenates at 15,000 rpm for 3 min. The renal tissue supernatant was used to evaluate the activity of ALT, AST, LDH, as well as creatinine and protein concentrations.

### 4.15. Statistical Analysis

The test results are shown as mean ± SEM (standard error mean). The parameters between the groups were compared by one-way analysis of variance (ANOVA) followed by post hoc Bonferroni test for means comparison (n = 10 for each group) [55]. STATISTICA software version 13.1 (StatSoft, Cracow, Poland) was used. Values of *p* < 0.05 were considered statistically significant.

## 5. Conclusions

We do not recommend the addition of Zn(II) (1 µg/L) and Zn(II) (1 µg/L) + PRL (0.1 µg/L) to the Biolasol solution.

## Figures and Tables

**Table 1:** The composition and functions of the individual components of Biolasol solution.

Component		Effect
K^+^	10 mmol/L	Electrolytes
Na^+^	105 mmol/L	Electrolytes
Ca^2+^	0.5 mmol/L	Electrolytes
Mg^2+^	5 mmol/L	Electrolytes
Cl^−^	10.5 mmol/L	Electrolytes
Dextran 70	0.7 g/L	Colloids
HCO_3_^−^	5 mmol/L	Buffers
Citrate Glucose	30 mmol/L 167 mmol/L	Impermeant Impermeant Energy substrates
Di-sodium edetate Fumarate	5 mmol/L 5 mmol/L	Additives Additives
Ascorbic acid	0.5 mmol/L	Antioxidant
pH	7.4	
Viscosity (cP)	2.90	
Osmolality (mOsm/kg H_2_O)	330	

## Data Availability

The data presented in this study are available on request from the corresponding author.

## Sample Availability

Samples of the compounds the Biolasol/modified Biolasol solution are available from the authors.
